# Laparoscopic *Versus* Robotic Completely Intracorporeal Jejunal Pouch Reconstruction After Gastrectomy: A Single-Center Analysis from Germany

**DOI:** 10.3390/cancers17162690

**Published:** 2025-08-19

**Authors:** Ani K. Stoyanova, Fiona Speichinger, Ioannis Pozios, Katharina Beyer, Ann-Kathrin Berg

**Affiliations:** 1 The Department of General and Visceral Surgery, Charité—Universitätsmedizin Berlin, Corporate Member of Freie Universität Berlin, Humboldt—Universität zu Berlin and Berlin Institute of Health (BIH), Hindenburgdamm 30, 12203 Berlin, Germany; fiona.speichinger@charite.de (F.S.); ioannis.pozios@charite.de (I.P.); ann-kathrin.berg@charite.de (A.-K.B.); 2 The Department of General, Visceral and Transplantation Surgery, Faculty of Medicine, University of Augsburg, 86159 Augsburg, Germany

**Keywords:** gastrectomy, gastric cancer, robotic surgery, laparoscopy, pouch reconstruction

## Abstract

**Background**: Gastric cancer is increasingly being diagnosed at early stages, enabling the application of curative oncological and surgical approaches. With the growing adoption of minimally invasive techniques, robotic surgery is gaining increasing prominence in the operating rooms. As described by Stoyanova et al., the robotic completely intracorporeal jejunal pouch reconstruction after gastrectomy offers potential benefits, including technical feasibility without significant intraoperative challenges or prolonged operative times, as well as long-term advantages such as a reduced incidence of midline incision hernias. **Objectives**: This retrospective, single-center study is the first to compare the clinical and oncological outcomes after laparoscopic *versus* robotic completely intracorporeal jejunal pouch reconstruction following gastrectomy. **Methods**: A total of 27 patients who underwent gastrectomy between 2018 and 2025 were included in the study, and were divided into two groups: 12 patients in the robotic and 15 patients in the laparoscopic group. The study evaluated mean operative time, intraoperative and postoperative complications, length of hospital and ICU stay, and certain oncological outcomes. **Results**: A main purpose of the robotic method is the avoidance of an unfavourable midline incision due to the completely intracorporeal pouch reconstruction without substantial technical or clinical disadvantages. **Conclusions**: Further research involving larger patient cohorts and extended follow-up periods is necessary to draw more definitive conclusions about the relative advantages of this surgical technique.

## Simple Summary

This retrospective single-center analysis compares the clinical and oncologic outcomes of laparoscopic *versus* robotic completely intracorporeal pouch reconstruction after gastrectomy in patients with gastric cancer. We suggest that the robotic method offers long-term benefits by avoiding a midline incision and consequently reducing the incidence of ventral hernias without technical surgical or oncologic disadvantages.

## 1. Introduction

Gastric cancer is the fifth most common malignancy and the fourth leading cause of cancer-related deaths worldwide [1]. An increasing number of patients are being diagnosed at earlier stages, enabling the use of curative surgical therapies. Robotic surgery is rapidly expanding globally and is widely regarded as a key development in the field of visceral surgery. It has been shown to be beneficial for patients as well as surgeons since it offers distinct advantages for both, including articulated robotic arms, enhanced 3D visualization, and superior lighting [2]. Previous studies have shown that robotic gastrectomy is associated with reduced intraoperative blood loss, faster postoperative bowel recovery, and fewer severe surgical complications overall [3,4]. Improved ergonomics and tremor elimination also contribute to greater comfort and precision for the operating surgeon [2].

There are numerous common techniques for reconstruction of a gastric reservoir after gastrectomy [5]. The Hunt-Lawrence pouch reconstruction has been shown to provide an overall improved quality of life for patients undergoing gastrectomy due to larger reservoir capacity and decreased occurrence of dumping syndrome [6].

The primary objective of this study is to compare robotic and laparoscopic gastrectomy, evaluating whether the robotic approach presents any significant clinical or oncological disadvantages—specifically regarding intraoperative and postoperative outcomes and lymph node yield [7]. On the contrary, as a result of performing a Pfannenstiel incision for the robotic completely intracorporeal pouch reconstruction instead of a mini-laparotomy in the laparoscopic patient group, we can potentially minimize the occurrence of midline incision hernias [8,9].

The aim of this study was to compare clinical and oncologic outcomes between laparoscopic and robotic approaches for completely intracorporeal Hunt-Lawrence pouch reconstruction following gastrectomy. We sought to assess the feasibility, safety, and potential advantages of the robotic technique in a single-center setting.

## 2. Materials and Methods

The robotic gastrectomy with completely intracorporeal jejunal pouch reconstruction was performed as described in detail by Stoyanova et al. using the DaVinci X robotic surgical system (Intuitive) [10]. Briefly summarized, the patient was placed in a supine French position, and the surgeon operated from the robotic console. Four robotic trocars and one assistant trocar were placed in the upper abdomen. A key distinguishing feature of the robotic approach was the completely intracorporeal reconstruction of the jejunal Hunt-Lawrence pouch and specimen retrieval via a Pfannenstiel incision, avoiding a midline laparotomy. The esophagojejunostomy was performed using a circular stapler inserted through the Pfannenstiel access site, and the jejunojejunostomy was completed intracorporeally using linear staplers. In contrast, the laparoscopic group required a mini-laparotomy for extracorporeal pouch construction and specimen retrieval through a midline incision.

**Figure 1:**
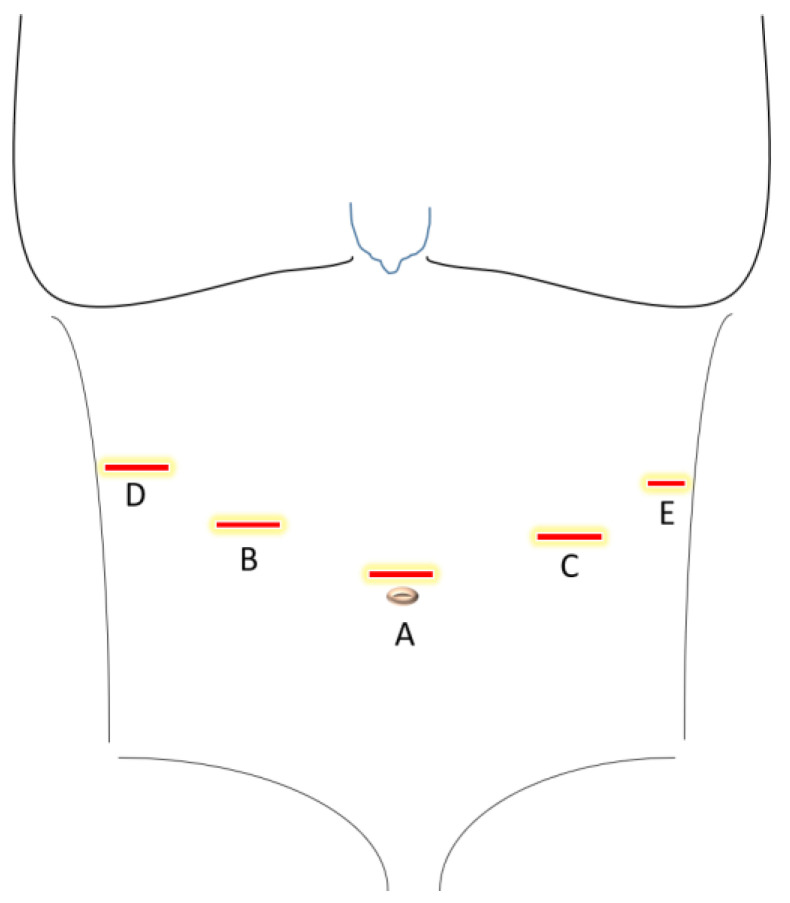
Trocar placement for laparoscopic gastrectomy with pouch reconstruction. A. Camera port (12 mm). B. and C. Operator trocars (12 mm). D. Liver paddle trocar (12 mm). E. Assistant trocar (5 mm).

The laparoscopic gastrectomy has been performed as follows [6,11]:

1. Patient in French position. The surgeon stands between the patient’s legs. First assistant stands on the left side of the patient, second assistant stands on the right side.
2. Port trocar placement:
  - supraumbilical incision, insertion of an optical trocar and establishment of pneumoperitoneum
  - intraabdominal inspection for exclusion of signs of peritoneal carcinosis as well as obvious liver metastasis
3. Main and assistant trocar placement: three 12 mm and one 5 mm trocars in the upper abdomen as described in Figure 1.
4. Mobilization of the greater omentum from the transverse colon.
5. Isolation and ligation of the left gastroepiploic vessels near the splenic vessels using Hem-o-lok clips.
6. Dissection of the short gastric vessels near the spleen.
7. Mobilization of the gastric fundus and exposure of the left diaphragmatic crus.
8. Lymphadenectomy along the splenic artery (lymph node (LN) stations 10 and 11).
9. Mobilization of the hepatic flexure and duodenum.
10. Ligation of the right gastroepiploic vessels (LN station 6).
11. Suprapyloric dissection:
  - liver retraction with a liver paddle. Incision in the lesser omentum and exclusion of an aberrant liver artery. Mobilization of the abdominal esophagus.
  - lymphadenectomy along the hepatic artery, left gastric artery, and celiac trunk (LN stations 7 and 9).
  - completion of the lymphadenectomy along the splenic artery (LN station 11) and extension of the dissection to the hepatoduodenal ligament (LN stations 5, 8, and 12).
12. Ligation of the coronary gastric vein (LN station 8).
13. Stapling of the duodenum 2 cm post pylorus.
14. Ligation of the left gastric artery (LN station 2).
15. Tying of the esophagus with a Mersilene band.
16. Specimen retrieval:
  - mini-laparotomy and insertion of an Alexis retractor. Division of the esophagus at the abdominal portion and specimen retrieval. Frozen section for conformation of tumor-free margins.
17. Cholecystectomy.
18. Reconstruction:
  - a 20 cm long Roux jejunal limb is used for the establishment of a 15 cm long Hunt-Lawrence pouch.
  - End-to-side esophagojejunostomy using a 29 mm circular stapler.
  - Side-to-side jejunojejunostomy 45 cm distal to the esophagojejunostomy for the biliary limb (Roux-en-Y reconstruction).
19. Final steps:
  - check for hemostasis and inspection of the anastomoses.
  - placement of a drain at the esophagojejunostomy.
  - fascia and wound closure.

We performed a retrospective analysis of 27 patients divided into two groups (15 patients in the laparoscopic group *versus* 12 patients in the robotic group). Patient allocation to either the laparoscopic or robotic group was not based on preoperative risk stratification or clinical characteristics. Instead, allocation was determined primarily by logistical factors, including surgeon availability, access to the robotic surgical system, and the presence of a specialized operating room team trained in robotic procedures, but also by patient’s preference and conviction. No formal randomization or matching protocol was applied. Patient demographics, mean operative time, length of ICU and in-hospital stay were evaluated, as well some oncological aspects such as tumor entity, the employment of neoadjuvant therapy, and the number of harvested lymph nodes. Furthermore, we compared intra- and postoperative outcomes, specifically wound infections, pulmonary complications, need of reintubation, need of re-resection due to positive specimen margins, and occurrence of anastomotic leakage. The operations were performed at a single center exclusively by two upper gastrointestinal surgeons, both of whom have undergone extensive training in laparoscopic and robotic surgery. Patients with BMI under 18 were excluded from the analysis. Due to the retrospective and exploratory nature of this study, no single primary endpoint was pre-specified. Instead, multiple clinical, operative, and oncologic outcomes were assessed, including the occurrence of severe postoperative complications (Clavien–Dindo grade ≥ III), mean operative time, hospital and ICU length of stay, and lymph node yield. All other variables are presented in the Results section according to their distribution with median and interquartile range or absolute and relative frequencies for each surgical group. These outcomes were chosen to provide an initial overview of the feasibility and safety of the completely intracorporeal robotic pouch reconstruction technique.

## 3. Results

### 3.1. Patient Demographics

Baseline characteristics for all cases are depicted in Table 1. Both groups were similar in age: 62.2 (±15.0) years in the laparoscopic group and 64.4 (±9.0) years in the robotic group. As expected, we had a predominantly male population in both groups (60% in the laparoscopic *versus* 66.7% in the robotic group). Body mass index was similar in both groups.

### 3.2. Operative Outcomes

We evaluated mean operating time (MOT), intra- and postoperative complications, pulmonary complications, need of reintubation, as well as length of ICU (intensive care unit) and overall hospital stay (Table 2). In each group, we observed one intraoperative complication, respectively. These complications were an injury of the diaphragm and an injury of the portal vein, respectively. Both complications were managed properly without serious issues for the patients in the further treatment course. As major postoperative complications, we considered each complication that was graded above Clavien–Dindo III and required intervention or re-operation. Such events included extended pleural effusions, which required drainage; acute respiratory distress syndrome (ARDS); chylous ascites; or anastomotic leakage. We have observed more major postoperative complications in the laparoscopic group (8/15 *versus* 3/12). There was one mortality case in the robotic group, which occurred on the eighth postoperative day due to septic shock and fulminant liver failure. Two cases of anastomotic leakage were observed in the laparoscopic group *versus* one in the robotic group. The patients in the robotic group spent less time in the ICU (1.9 ± 2.7 *versus* 4.5 ± 9.6), as well as less overall time in the hospital (15.8 ± 13.5 *versus* 21.8 ± 18.2 days). Pulmonary complications and need of reintubation were similar in both groups. No wound infections were observed in both groups.

### 3.3. Oncologic Aspects

We compared tumor entities, reception of neoadjuvant therapy, surgical margin status, and number of harvested lymph nodes (Table 3). In the laparoscopic group, in one patient, there was no tumor detected in the specimen pathology evaluation, even though a preoperative biopsy confirmed adenocarcinoma. One patient had both tumor entities: adeno- and signet cell carcinoma, and one patient had a pleomorphic rhabdomyosarcoma. 13 patients (86.7%) in the laparoscopic *versus* 9 (75%) in the robotic group received neoadjuvant chemotherapy. The numbers of harvested lymph nodes were similar in both groups (35.27 ± 12.3 *versus* 35.33 ± 7.3).

Furthermore, we compared tumor entities, reception of neoadjuvant therapy, surgical margin status, and number of harvested lymph nodes (Table 4). Disease-free gross margins (R2) were observed for all patients in both groups. The frozen section procedure during operation showed positive microscopic margins (R1) in two cases in the robotic group and a re-resection was performed in both patients.

## 4. Discussion

In this retrospective, single-center study, we demonstrate that robotic gastrectomy is not inferior to the well-established laparoscopic technique in terms of intraoperative challenges, postoperative complications, and key oncologic metrics such as lymph node retrieval. Moreover, we are taking into consideration the potential benefit of a Pfannenstiel incision to reduce the occurrence of midline incisional hernias resulting from the completely intracorporeal pouch reconstruction [8,9]. This study was exploratory and not sufficiently powered to assess any single predefined endpoint. Instead, we reported a range of clinically relevant outcomes to provide preliminary insights into the robotic intracorporeal pouch reconstruction technique. These findings are intended to generate hypotheses for future prospective studies.

Robotic surgery is gaining increasing attention in both surgical and oncologic fields due to its technical advantages. The articulated joints of robotic instruments allow access to anatomically complex areas that are often difficult to reach with rigid laparoscopic tools. Enhanced lighting, three-dimensional visualization, tremor filtration, and improved ergonomics contribute to a more favorable working environment for both surgeons and their assistants [12,13]. However, the widespread adoption of robotic systems also has several limitations, including high acquisition and maintenance costs, the need for specialized training, and sometimes prolonged operative times. Despite these challenges, robotic systems are being rapidly integrated into surgical departments worldwide. This trend underscores the need for rigorous evaluation of their benefits over traditional laparoscopic and open techniques, particularly in the context of surgical oncology, where robotic platforms are being increasingly explored for their potential to improve patient outcomes.

Beyond oncologic efficacy, optimizing long-term quality of life remains a crucial goal in the multidisciplinary care of patients undergoing gastrectomy. Historically, total gastrectomy without pouch reconstruction has been closely associated with substantial weight loss, malnutrition, and both early and late dumping syndromes [14]. In particular, prophylactic total gastrectomy in young patients with CDH1 gene mutations has been considered a highly invasive intervention due to its profound impact on nutritional status and overall well-being [15,16].

There is emerging evidence that the established safety resection margins for gastric cancer are overestimated and that shorter resection distance may still result in favorable oncologic outcomes. For tumors located in the distal part of the stomach, a resection margin of 8 cm for diffuse and 5 cm for intestinal adenocarcinoma of the stomach has been the standard for decades and is still adopted by most surgical guidelines. In this study, two robotic group patients required intraoperative re-resection due to positive microscopic margins (R1), as identified via frozen section. The need for intraoperative re-resection in two robotic cases due to R1 margins highlights the importance of real-time pathology and surgical precision. While re-resection achieved R0 margins, the incidence raises questions regarding margin assessment and technique during robotic procedures. A large-scale study from South Korea demonstrated that the different resection distances have no impact on the overall survival as long as an R0 is being achieved [17]. The US Gastric Cancer Collaborative provided evidence that the intraoperative conversion of R1 to R0 through re-resection after frozen section procedure does not impact the recurrence-free or overall survival of patients with gastric cancer [18,19]. Based on these findings, surgical strategies increasingly focus on preserving the gastric fundus whenever oncologically appropriate, as this may provide reservoir function and improve postoperative quality of life. This trend also partly explains the limited sample size in our study, as total gastrectomy was not required in many patients due to the feasibility of gastric preservation. However, total gastrectomy remains necessary for advanced-stage tumors or those located in the upper or middle third of the stomach [20]. Several reconstruction techniques are available following total gastrectomy. The ideal method should ensure adequate reservoir capacity and nutritional intake while minimizing the risk of dumping syndrome to support long-term quality of life. Beyond traditional Billroth-I and Billroth-II techniques for distal gastrectomy, the Roux-en-Y reconstruction has become the standard after total gastrectomy due to its ability to reduce bile reflux and provide satisfactory nutritional outcomes [21]. A disadvantage of this method is the risk of Roux-stasis syndrome with delayed gastric emptying [22]. Jejunal pouch reconstruction, though technically more demanding, offers several advantages including a larger reservoir capacity, reduced rates of dumping and reflux, and improved nutritional intake—without a notable risk of Roux stasis [23]. At our center, we prefer the Hunt–Lawrence jejunal pouch reconstruction, using a 20 cm Roux limb to create a 15 cm pouch. The proximal anastomosis is incompletely stapled to enhance blood supply and reduce the risk of anastomotic leakage [24].

Selection bias remains a relevant limitation in our retrospective study design. Patients were not randomized, and group assignment was influenced by logistical considerations such as availability of the robotic system, surgeon schedules, and the presence of a dedicated robotic surgical team. These factors may have contributed to improved operative coordination and outcomes in the robotic group. Additionally, although the BMI difference between groups was small (24.8 vs. 26.25 kg/m^2^), such differences may impact outcomes in small cohorts. These limitations are acknowledged and further emphasize the need for future prospective studies incorporating randomization or propensity score matching to better control for confounding variables.

Furthermore, a potential influence of the learning curve in robotic surgery must be acknowledged. All operations in this study were exclusively performed by two experienced upper gastrointestinal surgeons who have undergone extensive training in laparoscopic and robotic visceral oncological surgery. However, variability in outcomes may still reflect the natural progression of proficiency with this newer technique. It should also be noted that the robotic technique was predominantly performed in the last few years of the study. Prior to that, the primary surgeon who performed the majority of the laparoscopic operations had already gained substantial expertise in minimally invasive upper gastrointestinal surgery. Furthermore, due to growing interest in robotic surgery, patients have increased their willingness and desire to undergo robotic surgery instead of laparoscopic surgery. Naturally, combining these two factors lead to an obvious bias that could be only avoided by conducting a large-scale randomized study.

While intraoperative complications were similar between groups, the robotic group had fewer major postoperative complications. This could suggest a trend toward improved safety; however, the small sample size limits the ability to draw statistically significant conclusions. A single mortality case occurred in the robotic group. The patient died on postoperative day 8 due to septic shock and fulminant liver failure. The patient had initially shown an unremarkable postoperative course but developed acute respiratory symptoms followed by rapid clinical deterioration and had to be readmitted to the ICU. Notably, imaging and re-laparoscopy excluded anastomotic leakage or other technical errors related to the robotic procedure. The patient’s clinical course was complicated by pleural empyema and pulmonary embolism, while no intraoperative technical issues were identified. Although this outcome was not attributed to the surgical method itself, it underscores the need for close postoperative monitoring and highlights the complexity of managing patients undergoing total gastrectomy. Lymph node yield was comparable between groups, meeting oncologic standards. Interestingly, mean operative time was lower in the robotic group (275.5 ± 63.7 min) compared to the laparoscopic group (329.8 ± 79.2 min) despite the complexity of intracorporeal reconstruction. This may reflect improved team coordination and the technical benefits of the robotic platform, although the role of selection bias cannot be excluded. The mean BMI was slightly lower in the robotic group (24.8 *versus* 26.25 kg/m^2^), and robotic surgeries were supported by specialized staff. These factors may have influenced outcomes and introduce confounding. Larger randomized trials, ideally with propensity matching and multivariate analysis, are needed to validate these preliminary findings. Due to the small sample size, the descriptive statistics cannot identify significance. A propensity score matching or multivariate analysis on properly sized cohorts are needed in future studies.

Ventral incisional hernias are not uncommon long-term complications in the field of visceral surgery. They are a relevant long-term complication of midline laparotomy, occurring in up to 20% of patients [25]. Specialized hernia experts advise strongly against use of midline incisions, even for the minimally invasive surgery [26]. Multiple long-term studies provide evidence that intracorporeal techniques and the avoidance of midline incision reduce the risk of midline incisional hernias substantially [27,28,29,30,31]. With the improved overall survival of gastric cancer patients due to advanced diagnostics and therapeutic concepts, this is an important topic that should be further evaluated.

Despite encouraging observations, our study has important limitations. These include its retrospective design, absence of systematic randomization, small sample size, and lack of statistical power. Therefore, the findings should be interpreted as exploratory and hypothesis-generating. Future research should focus on prospective, multicenter trials with larger patient cohorts, extended follow-up, quality-of-life measures, and cost-effectiveness analysis.

## 5. Conclusions

This retrospective, single-center study compared laparoscopic and robotic approaches to completely intracorporeal Hunt-Lawrence pouch reconstruction following gastrectomy in patients with gastric cancer. No clear superiority was observed between the two methods in terms of intraoperative, clinical, or oncologic outcomes. In addition, based on previous studies, we propose that the Pfannenstiel incision may be an effective strategy to reduce the risk of ventral incisional hernias. However, further studies with larger patient cohorts and extended follow-up periods are needed to validate these findings and better inform clinical practice.

## Figures and Tables

**Table 1:** Patient demographics.

	Laparoscopic (*n* = 15)	Robotic (*n* = 12)
Age (yr)	62.2 ± 15.0	64.4 ± 9.0
Gender male (n/%)	9/60%	8/66.7%
Body mass index (kg/m^2^)	26.25 ± 4.97	24.8 ± 4.19
Regular alcohol consumption	3/20%	2/16.7%
Smokers	2/13.3%	3/25%

**Table 2:** Operative outcomes and complications.

	Laparoscopic (*n* = 15)	Robotic (*n* = 12)
MOT (minutes)	329.8 ± 79.21	275.45 ± 63.74
Intraoperative complications	1	1
Major postoperative complications	8	3
Pulmonary complications	6	5
Reintubation	2	1
Length of ICU stay (days)	4.5 ± 9.6	1.9 ± 2.7
Length of hospital stay (days)	21.8 ± 18.2	15.8 ± 13.5
Anastomotic leakage	2	1
Wound infections	0	0
Mortality	0	1

**Table 3:** Oncologic aspects and outcomes.

	Laparoscopic (*n* = 15)	Robotic (*n* = 12)
adenocarcinoma	9	10
signet ring carcinoma	3	2
other *	3	0
neoadjuvant therapy	13	9
number of harvested lymph nodes	35.27 ± 12.3	35.33 ± 7.3
re-resection needed	0	2

* One patient had partially adenocarcinoma and signet cell carcinoma, one had pleomorphic rhabdomyosarcoma, and one patient had no detectable tumor cells.

**Table 4:** Pathologic and nodal stages.

		Laparoscopic (*n* = 15)	Robotic (*n* = 12)
Pathologic T-stage	pT0 pTis pT1 pT2 pT3 pT4	1 0 2 0 9 3	0 0 1 3 5 3
Pathologic nodal status (pN)	pN0 pN1 pN2 pN3	10 1 1 3	6 1 4 1
Disease-free gross margins (R2) Disease-free microscopic margins (R1)		15 15	12 10

## Data Availability

The original contributions presented in this study are included in the article. Further inquiries can be directed to the corresponding authors.

## Abbreviations

The following abbreviations are used in this manuscript:

3D	three-dimensional
ARDS	Acute respiratory distress syndrome
BMI	Body mass index
CDH1	Cadherin-1 gene
ICU	Intensive care unit
EA	Ethics approval code
MOT	Mean operating time
LN	Lymph node
pN	Pathologic nodal Stage
pT	Pathologic tumor Stage
R0	Complete tumor resection with negative margins
R1	Microscopic residual tumor
R2	Macroscopic residual tumor
yr	years
